# Micro-DeMix: a mixture beta-multinomial model for investigating the heterogeneity of the stool microbiome compositions

**DOI:** 10.1093/bioinformatics/btae667

**Published:** 2024-11-19

**Authors:** Ruoqian Liu, Yue Wang, Dan Cheng

**Affiliations:** School of Mathematical and Statistical Sciences, Arizona State University, Tempe, AZ 85251, United States; Department of Biostatistics and Informatics, Colorado School of Public Health, Aurora, CO 80045, United States; School of Mathematical and Statistical Sciences, Arizona State University, Tempe, AZ 85251, United States

## Abstract

**Motivation:**

Extensive research has uncovered the critical role of the human gut microbiome in various aspects of health, including metabolism, nutrition, physiology, and immune function. Fecal microbiota is often used as a proxy for understanding the gut microbiome, but it represents an aggregate view, overlooking spatial variations across different gastrointestinal (GI) locations. Emerging studies with spatial microbiome data collected from specific GI regions offer a unique opportunity to better understand the spatial composition of the stool microbiome.

**Results:**

We introduce Micro-DeMix, a mixture beta-multinomial model that deconvolutes the fecal microbiome at the compositional level by integrating stool samples with spatial microbiome data. Micro-DeMix facilitates the comparison of microbial compositions across different GI regions within the stool microbiome through a hypothesis-testing framework. We demonstrate the effectiveness and efficiency of Micro-DeMix using multiple simulated datasets and the inflammatory bowel disease data from the NIH Integrative Human Microbiome Project.

**Availability and implementation:**

The R package is available at https://github.com/liuruoqian/MicroDemix.

## 1 Introduction

The human gut microbiome and its role in host health have been the subject of extensive research, establishing its involvement in human metabolism (Devaraj *et al.* 2013), nutrition (Zhang 2022), physiology (Andoh 2016), and immune function (Wu and Wu 2012, Bull and Plummer 2014). Recent studies have also demonstrated the association between the gut microbiota and the emergence of obesity (Aoun *et al.* 2020), metabolic syndrome and the onset of type 2 diabetes (Devaraj *et al.* 2013, Iatcu *et al.* 2021). Moreover, altered composition and function of the gut microbiota has been found associated with chronic diseases (Pellanda *et al.* 2021) ranging from gastrointestinal (GI) inflammatory (Shan *et al.* 2022) and metabolic conditions to neurological (Cryan *et al.* 2020), cardiovascular (Tang *et al.* 2017), and respiratory illnesses (Durack and Lynch 2019, Chunxi *et al.* 2020).

Most scientific findings regarding the gut microbiome have been derived from stool samples. However, it is increasingly recognized that the stool microbiome offers only an aggregate view, overlooking the spatial heterogeneity of the microbiome across different GI locations (Ahn *et al.* 2023, Levitan *et al.* 2023). Recent studies have revealed significant variations in microbial composition and function along distinct segments of the GI tract (Leite *et al.* 2020). For instance, the small intestine is dominated by Lactobacillaceae and Enterobacteriaceae, whereas the colon harbors species from families such as Bacteroidaceae, Prevotellaceae, Rikenellaceae, Lachnospiraceae, and Ruminococcaceae (Donaldson *et al.* 2016). Furthermore, spatial metagenomics applied to the mouse colonic microbiome has revealed a heterogeneous distribution of taxa throughout the gut (Sheth *et al.* 2019). Thus, understanding the spatial composition of the stool microbiome is essential for enhancing the biological relevance of stool-based microbiome analyses, allowing for more accurate interpretations of its role in health and disease. Although spatial microbiome data from specific GI locations remain relatively rare due to the invasive nature of sample collection, they offer a unique opportunity to deconvolute stool samples, thereby providing a deeper understanding of the gut microbiome’s spatial heterogeneity.

We develop Micro-DeMix, a novel statistical model designed to deconvolute the fecal microbiome into specific GI locations by integrating spatial microbiome data. The development of Micro-DeMix was motivated by data from the inflammatory bowel disease (IBD) Multiomics Database (IBDMDB) project, part of the NIH Integrative Human Microbiome Project (iHMP). This project followed 132 individuals and collected 1785 stool samples and 651 intestinal biopsies over time (Integrative HMP *et al.* 2019). The integrated nature of this dataset, combining microbial profiles from both stool samples and distinct GI locations, highlighted the need for a tool like Micro-DeMix to effectively analyze and integrate these diverse sources of microbiome data.

Micro-DeMix is a mixture beta-multinomial model developed to deconvolute the fecal microbiome into specific GI locations. The multinomial component effectively handles the compositional nature of microbiome data by jointly modeling microbial proportions, while the beta component adjusts for sample heterogeneity, incorporating key demographic and clinical variables that may influence the composition of the fecal microbiome. We introduce two estimation procedures for the parameters in the Micro-DeMix model. The first procedure directly maximizes the likelihood of the observed microbial counts, offering computational efficiency. This approach is also equipped with an efficient hypothesis-testing framework for comparing microbial compositions from different GI regions within the mixed stool sample based on the asymptotic null distribution. The second procedure is based on the Expectation-Maximization (EM) algorithm, which provides greater numerical robustness than the first method. It is particularly useful for scenarios with extremely rare microbes, where the first procedure may encounter limitations, although it is computationally more intensive. This EM approach includes a permutation test to identify differentially abundant microbial groups across GI locations. Unlike conventional differential abundance (DA) tools, which compare microbial abundances between predefined groups (e.g. disease versus healthy or across body sites), Micro-DeMix is specifically designed to detect compositional changes within a mixed population (e.g. the stool sample), where the groups are not prespecified and must be statistically estimated. This enables Micro-DeMix to uncover group differences and spatial heterogeneity within the stool microbiome by integrating spatial microbiome data. Traditional DA tools, which rely on predefined group distinctions, are less suited to detect these types of within-sample variations. We demonstrate the effectiveness of Micro-DeMix through extensive simulation studies and an application to IBD cohorts, where we integrate stool microbiome data with rectum microbiome data.

The rest of the paper is organized as follows. In Section 2, we describe the Micro-DeMix model, develop two maximum-likelihood-based estimation procedures, and propose hypothesis-testing frameworks for detecting differentially abundant microbes between GI locations. In Section 3, we conduct simulation studies to demonstrate the effectiveness of Micro-DeMix in terms of the estimation accuracy and the power of the hypothesis testing method. In Section 4, we apply our method to the IBD microbiome data from iHMP to elucidate the composition of fecal microbiome in IBD patients. We close with a discussion of our method in Section 5.

## 2 Materials and methods

### 2.1 The Micro-DeMix model

In this section, we present a beta-multinomial framework for modeling microbial abundance in stool samples. Our model considers a multivariate extension of the beta-binomial model proposed in a recent research (Martin *et al.* 2020) which models individual taxa separately. As shown later, while this multivariate extension allows for joint modeling of multiple microbes, it also brings several new statistical and computational challenges.

For ease of presentation and biological relevance, we assume the reference spatial microbiome data are collected at the rectum. However, the methodology proposed below can seamlessly extend to other segments of the GI tract, including the ileum, jejunum, colon, and beyond, given the data availability. Let $y_{jg}^{\left(r\right)}$ denotes the observed absolute abundance for taxon *g* in the *j*th subject of the reference rectum microbiome data for $j=1,\ldots ,S^{\left(r\right)}$. Let $y_{ig}$ denotes the observed absolute abundance for taxon *g* in the *i*th stool samples for g=1,…,G and i=1,…,S. For all subjects, we also collect covariate information $x_{j}^{\left(r\right)}$ and $x_{i}$ for $j=1,\ldots ,S^{\left(r\right)}$ and i=1,…,S. We model the stool microbial abundance $y_{i}={\left(y_{i1},\ldots ,y_{iG}\right)}^{⊺}$ using a multinomial model $y_{i}|p_{i}∼\text{Multinomial}(N_{i},p_{i}),$ where $N_{i}=\sum_{g=1}^{G}y_{ig}$ and $p_{i}={\left(p_{i1},\ldots ,p_{iG}\right)}^{⊺}$ is the underlying true microbial proportions. We are particularly interested in the spatial composition of the stool microbiome. Acknowledging that the stool microbiome consists of microbes from rectum and other GI locations (mostly, the small intestine), we consider

(1) $$p_{ig}=\pi_{i}p_{g}^{\left(r\right)}+(1-\pi_{i})p_{g}^{\left(o\right)};$$

here, $\pi_{i}\in (0,1)$ denotes the latent proportion of the rectal microbiome in the ith stool sample; $p_{g}^{\left(r\right)}$ and $p_{g}^{\left(o\right)}$ denotes the proportion of taxon *g* in the rectum and other GI locations, respectively.

To allow $\pi_{i}$ to vary across individuals, we assume $\pi_{i}∼\text{Beta}(a_{1,i},a_{2,i}).$ Hence, we write the joint density function of ($y_{ig},\pi_{i}$) as

(2) $$\begin{array}{l} f(y_{ig},\pi_{i}|p_{g}^{\left(r\right)},p_{g}^{\left(o\right)},a_{1,i},a_{2,i})=\frac{N_{i}!}{y_{i1}!\ldots y_{iG}!}\prod_{g=1}^{G}\{\pi_{i}p_{g}^{\left(r\right)}\\ +(1-\pi_{i})p_{g}^{\left(o\right)}{\}}^{y_{ig}}\frac{\pi_{i}^{a_{1,i}-1}{\left(1-\pi_{i}\right)}^{a_{2,i}-1}}{B(a_{1,i},a_{2,i})}, \end{array}$$

where *B*(*a*_1,*i*_, *a*_2,*i*_) = Γ(*a*_1,*i*_)Γ(*a*_2,*i*_)/Γ(*a*_1,*i*_ + *a*_2,*i*_)

To capture the association between $\pi_{i}$ and the subjects’ characteristics, we further link each $\pi_{i}$ with covariates $x_{i}$ through a beta-regression model. With additional reparameterization

(3) $$\mu_{i}=\frac{a_{1,i}}{a_{1,i}+a_{2,i}},$$

(4) $$\begin{array}{c} \phi_{i}=a_{1,i}+a_{2,i}, \end{array}$$

we consider

(5) $$\text{log}(\frac{\mu_{i}}{1-\mu_{i}})=\beta_{0}+x_{i}^{⊺}\beta ,$$

(6) $$\begin{array}{c} \;\text{log}(\phi_{i})=\gamma_{0}+x_{i}^{⊺}\gamma . \end{array}$$

Some calculations yield that $\mu_{i}$ is the expected proportion of the rectum microbiome in the *i*th stool sample, i.e. $\mu_{i}=E(\pi_{i})$, and $\text{Var}(\pi_{i})=\mu_{i}(1-\mu_{i})/(1+\phi_{i})$, where $\phi_{i}$ is known as the precision parameter.

Thus, our first goal is to estimate each $p_{g}^{\left(r\right)}$ and $p_{g}^{\left(o\right)}$ to understand the spatial heterogeneity of the stool microbiome. Then, we aim to identify microbial groups that are differentially abundant between the rectum and other GI locations with the stool sample. Below we will present two procedures for these two analyses, each with its own strengths for different types of applications.

Remark 1. *Under the proposed model, we derive the mean and variance of* $y_{ig}$:

$$\begin{array}{c} E(y_{ig})=N_{i}\left[p_{g}^{\left(r\right)}\frac{a_{1,i}}{a_{1,i}+a_{2,i}}+p_{g}^{\left(o\right)}\left(1-\frac{a_{1,i}}{a_{1,i}+a_{2,i}}\right)\right]\\ \text{Var}(y_{ig})=N_{i}\{\frac{a_{1,i}}{a_{1,i}+a_{2,i}}(p_{g}^{\left(r\right)}-2p_{g}^{\left(r\right)}p_{g}^{\left(o\right)}-p_{g}^{\left(o\right)}+2{\left(p_{g}^{\left(o\right)}\right)}^{2})\\ +\left(\frac{a_{1,i}^{2}}{{\left(a_{1,i}+a_{2,i}\right)}^{2}}+\frac{a_{1,i}a_{2,i}}{{\left(a_{1,i}+a_{2,i}\right)}^{2}(a_{1,i}+a_{2,i}+1)}\right)\\ (-{\left(p_{g}^{\left(r\right)}\right)}^{2}+2p_{g}^{\left(r\right)}p_{g}^{\left(o\right)}-{\left(p_{g}^{\left(o\right)}\right)}^{2})+p_{g}^{\left(o\right)}-{\left(p_{g}^{\left(o\right)}\right)}^{2}\}\\ +N_{i}^{2}{\left(p_{g}^{\left(r\right)}-p_{g}^{\left(o\right)}\right)}^{2}\frac{a_{1,i}a_{2,i}}{{\left(a_{1,i}+a_{2,i}\right)}^{2}(a_{1,i}+a_{2,i}+1)}. \end{array}$$

*See derivations in the Supplementary Material.*

### 2.2 Micro-DeMix-1

#### 2.2.1 Estimation

The joint likelihood in Equation (2) involves $\pi_{i}$, which is unobserved. Thus, we calculate the log-likelihood of observing $\left\{y_{ig}:i=1,\ldots ,S;g=1,\ldots ,G\right\}$ in stool samples:

(7) $$\text{log}\;L(\theta )=\sum_{i=1}^{S}\;\text{log}\;\int_{0}^{1}f(y_{ig},\pi_{i}|\theta )d\pi_{i},$$

where $\theta ={\left(p^{\left(r\right)},p^{\left(o\right)},\beta_{0},\beta ,\gamma_{0},\gamma \right)}^{⊺}$. We estimate θ in two steps. We first estimate $p_{g}^{\left(r\right)}$ using the reference rectum microbiome data for g=1,…,G. Recall that $y_{jg}^{\left(r\right)}$ denotes the observed absolute abundance for taxon *g* and sample *j*, $j=1,\ldots ,S^{\left(r\right)}$, collected from rectum. Let $N_{j}^{\left(r\right)}$ denotes the sequencing depth for the *j*th sample from rectum. We assume the absolute abundance to follow a multinomial distribution

(8) $$y_{j}^{\left(r\right)}∼\text{Multinomial}(N_{j}^{\left(r\right)},p^{\left(r\right)}).$$

Based on this model, we estimate $p_{g}^{\left(r\right)}$ with the sample proportions

(9) $${\hat{p}}_{g}^{\left(r\right)}=\frac{\sum_{j}y_{jg}^{\left(r\right)}}{\sum_{j}N_{j}^{\left(r\right)}}.$$

After replacing $p_{g}^{\left(r\right)}$ in Equation (7) by ${\hat{p}}_{g}^{\left(r\right)}$, we estimate the remaining parameters using an iterative procedure with the main steps given in Algorithm 1.

Algorithm 1. Estimating $\left\{p_{g}^{\left(o\right)},\beta_{0},\beta ,\gamma_{0},\gamma \right\}$ for Micro-DeMix

**Require:**$y_{ig},x_{i}$, an integer M, a small number σ (e.g. σ=0.001)

1: **Initialize:**$\{\beta_{0},\beta ,\gamma_{0},\gamma \}={\left\{\beta_{0},\beta ,\gamma_{0},\gamma \right\}}^{0}$

2: **for**m=1,…,M**do**

3:      Update ${\left\{p_{g}^{\left(o\right)}\right\}}^{m}=\text{argmax}\;\text{log}\;L(\theta )$ as in (7) given   $\{\beta_{0},\beta ,\gamma_{0},\gamma \}={\left\{\beta_{0},\beta ,\gamma_{0},\gamma \right\}}^{m-1}$.

4:   Update ${\left\{\beta_{0},\beta ,\gamma_{0},\gamma \right\}}^{m}=\text{argmax}\;\text{log}\;L(\theta )$ as in (7) given   $\{p_{g}^{\left(o\right)}\}={\left\{p_{g}^{\left(o\right)}\right\}}^{m}$. Let $L_{m}$ denote the value of (7) at this step.

5:   **if**$\left|L_{m}-L_{m-1}\right|\leq \sigma$**then**

6:    **return**$\hat{\theta }={\left\{p_{g}^{\left(o\right)},\beta_{0},\beta ,\gamma_{0},\gamma \right\}}^{m}$

7:   **end if**

8: **end for**

9: **return**$\hat{\theta }={\left\{p_{g}^{\left(o\right)},\beta_{0},\beta ,\gamma_{0},\gamma \right\}}^{M}$

Specifically, in the *m*th iteration, we obtain ${\left\{p_{g}^{\left(o\right)}\right\}}^{m}$ by maximizing the objective function (7) given ${\left\{\beta_{0},\beta ,\gamma_{0},\gamma \right\}}^{m-1}$. Since the integral in Equation (7) does not have a closed form, we used the Gauss–Legendre quadrature (Golub and Welsch 1969) to approximate it. Solving for ${\left\{p_{g}^{\left(o\right)}\right\}}^{m}$ is a constrained optimization problem because $p_{g}^{\left(o\right)}\in [0,1]$ and $\sum_{g=1}^{G}p_{g}^{\left(o\right)}=1$. To address this problem, we consider the parameterization

(10) $$p_{g}^{\left(o\right)}=\frac{e^{u_{g}}}{\sum_{k=1}^{G}e^{u_{k}}},$$

where $u_{g}\in R$ for g=1,…,G. With this reparametrization, we first obtain ${\left\{u_{g}\right\}}^{m}$ using the Nelder–Mead optimization algorithm from R package optimx and then calculate ${\left\{p_{g}^{\left(o\right)}\right\}}^{m}$ from ${\left\{u_{g}\right\}}^{m}$ using Equation (10). Finally, given ${\left\{p_{g}^{\left(o\right)}\right\}}^{m}$, we obtain the optimal set ${\left\{\beta_{0},\beta ,\gamma_{0},\gamma \right\}}^{m}$ using the Nelder–Mead optimization algorithm by maximizing log-likelihood function (7). We repeat this iterative procedure until we achieve convergence or reach the maximum number of iterations.

#### 2.2.2 Hypothesis testing

The proposed Micro-DeMix framework also facilitates the investigation of the biodiversity of the fecal microbiome by detecting microbial groups that are differentially abundant in rectum and other GI locations. Specifically, we consider a hypothesis testing problem with $H_{0}:p^{\left(o\right)}=p^{\left(r\right)}$, where $p^{\left(o\right)}$ and $p^{\left(r\right)}$ are introduced in Section 2.1, representing the true proportions of the microbes of interest in the rectum and other GI locations, respectively.

Under $H_{0}$, we have $||p^{\left(o\right)}-p^{\left(r\right)}|{|}_{2}=0$, motivating the following test statistic:

(11) $$\hat{T}={\left({\hat{p}}^{\left(o\right)}-{\hat{p}}^{\left(r\right)}\right)}^{⊺}({\hat{p}}^{\left(o\right)}-{\hat{p}}^{\left(r\right)}),$$

where ${\hat{p}}^{\left(o\right)}$ are maximum likelihood estimators obtained from Algorithm 1 in Section 2.2, and ${\hat{p}}^{\left(r\right)}$ is given in Equation (9). Under $H_{0}:p_{g}^{\left(r\right)}=p_{g}^{\left(o\right)}$, we can rewrite Equation (11) as

(12) $$\begin{array}{c} \hat{T}=\sum_{g=1}^{G}{\left({\hat{p}}_{g}^{\left(r\right)}-p_{g}^{\left(r\right)}\right)}^{2}+\sum_{g=1}^{G}{\left({\hat{p}}_{g}^{\left(o\right)}-p_{g}^{\left(o\right)}\right)}^{2}\\ -2\sum_{g=1}^{G}\left({\hat{p}}_{g}^{\left(r\right)}-p_{g}^{\left(r\right)}\right)({\hat{p}}_{g}^{\left(o\right)}-p_{g}^{\left(o\right)}), \end{array}$$

indicating that $\hat{T}$ is a function of ${\hat{p}}^{\left(r\right)}-p^{\left(r\right)}$ and ${\hat{p}}^{\left(o\right)}-p^{\left(o\right)}$ under $H_{0}$. Thus, finding the null distribution of $\hat{T}$ reduces to finding the null distributions of ${\hat{p}}^{\left(r\right)}-p^{\left(r\right)}$ and ${\hat{p}}^{\left(o\right)}-p^{\left(o\right)}$. Specifically, applying the central limit theorem (CLT) based on model (8), we have

(13) $$\sqrt{N^{\left(r\right)}}({\hat{p}}^{\left(r\right)}-p^{\left(r\right)})\overset{d}{\to }N_{G}(0,V(p^{\left(r\right)})),$$

where

$$\begin{array}{c} V(p^{\left(r\right)})=\left(\begin{array}{cccc} p_{1}^{\left(r\right)}(1-p_{1}^{\left(r\right)}) & -p_{1}^{\left(r\right)}p_{2}^{\left(r\right)} & \ldots & -p_{1}^{\left(r\right)}p_{G}^{\left(r\right)}\\ -p_{1}^{\left(r\right)}p_{2}^{\left(r\right)} & p_{2}^{\left(r\right)}(1-p_{2}^{\left(r\right)}) & \ldots & -p_{2}^{\left(r\right)}p_{G}^{\left(r\right)}\\ \vdots & & \ddots & \vdots \\ -p_{1}^{\left(r\right)}p_{G}^{\left(r\right)} & -p_{2}^{\left(r\right)}p_{G}^{\left(r\right)} & \ldots & p_{G}^{\left(r\right)}(1-p_{G}^{\left(r\right)}) \end{array}\right) \end{array}$$

and $N^{\left(r\right)}=\sum_{j=1}^{S^{\left(r\right)}}N_{j}^{\left(r\right)}$. Also, under $H_{0}$, the joint density function in Equation (2) reduces to

$$\begin{array}{c} f(y_{ig},\pi_{i}|p_{g}^{\left(o\right)},a_{1,i},a_{2,i})=\frac{N_{i}!}{y_{i1}!\ldots y_{iG}!}\prod_{g=1}^{G}{\left\{p_{g}^{\left(o\right)}\right\}}^{y_{ig}}\\ \;\frac{\pi_{1}^{a_{1,i}-1}{\left(1-\pi_{i}\right)}^{a_{2,i}-1}}{B(a_{1,i},a_{2,i})} \end{array}$$

and the density function (2) becomes

$$f(y_{ig}|\cdot )=\frac{N_{i}!}{y_{i1}!\ldots y_{iG}!}\prod_{g=1}^{G}{\left\{p_{g}^{\left(o\right)}\right\}}^{y_{ig}}\int_{0}^{1}\frac{\pi_{1}^{a_{1,i}-1}{\left(1-\pi_{i}\right)}^{a_{2,i}-1}}{B(a_{1,i},a_{2,i})}d\pi_{i}.$$

As the integral of the beta density is 1, we have

$$f(y_{ig}|p_{g}^{\left(o\right)},a_{1,i},a_{2,i})=\frac{N_{i}!}{y_{i1}!\ldots y_{iG}!}\prod_{g=1}^{G}{\left\{p_{g}^{\left(o\right)}\right\}}^{y_{ig}}.$$

This indicates that under $H_{0}$, the proposed beta-multinomial model in Section 2.1 reduces to a multinomial model. Indeed, when $p_{g}^{\left(r\right)}=p_{g}^{\left(o\right)}$, $\text{Var}(y_{ig})$ in Remark 1 simplifies to $N_{i}p_{g}^{\left(o\right)}(1-p_{g}^{\left(o\right)})$, which equals the variance of $y_{ig}$ under the multinomial model. Thus, under $H_{0}$, the maximum likelihood theory (MLE) derived from the mixture beta-multinomial model is equivalent to that derived from the multinomial model. Thus, we can apply the CLT again and obtain

(14) $$\sqrt{N}({\hat{p}}^{\left(o\right)}-p^{\left(o\right)})\overset{d}{\to }N_{G}(0,V(p^{\left(r\right)})),$$

where $N=\sum_{i=1}^{S}N_{i}$ is the total counts of all samples collected from stool.

Assuming no overlapping between stool samples and rectum samples, we establish the asymptotic distribution of $\hat{T}$ in the following result.

Proposition 1. *Let random vector* $\left(y_{1},y_{2}\right)$*follow the multivariate normal distribution*

$$\begin{array}{c} N_{2G}\left(\begin{array}{ccc} \left(\begin{array}{c} 0\\ 0 \end{array}\right) & , & \left(\begin{array}{cc} V(p^{\left(r\right)}) & 0\\ 0 & V(p^{\left(r\right)}) \end{array}\right) \end{array}\right). \end{array}$$

*Suppose* $p^{\left(o\right)}=p^{\left(r\right)}$*and*$N^{\left(r\right)}/N\to K>0$*. Then*$N\hat{T}\overset{d}{\to }g(y_{1},y_{2})$*as*N→∞*, where*$g(y_{1},y_{2})=y_{1}^{⊺}y_{1}+(1/K)y_{2}^{⊺}y_{2}-(2/\sqrt{K})y_{1}^{⊺}y_{2}$.

As a special case of Proposition 1, when K=1 or $N^{\left(r\right)}=N$, we have $N\hat{T}\overset{d}{\to }g(y_{1},y_{2})=y_{1}^{⊺}y_{1}+y_{2}^{⊺}y_{2}-2y_{1}^{⊺}y_{2}$. Based on Proposition 1, we develop a simulation-based algorithm for calculating the p-value, as detailed in Algorithm 2.

Algorithm 2. Simulation method of testing $H_{0}:p^{\left(o\right)}=p^{\left(r\right)}$

**Require:** $y_{jg}^{\left(r\right)},y_{ig},x_{i}$ , a large integer B (e.g. B=10 000)

1: Estimate ${\hat{p}}^{\left(o\right)}$ and ${\hat{p}}^{\left(r\right)}$ as in Section 2.2.

2: Compute $\hat{T}$ as in (11) using ${\hat{p}}^{\left(o\right)}$ and ${\hat{p}}^{\left(r\right)}$.

3: **for**b=1,…,B**do**

4:   Simulate vector $v_{1},v_{2}$ independently from  $N_{G}(0,V({\hat{p}}^{\left(r\right)}))$ in (13) and (14).

5:   Approximate ${\hat{p}}^{\left(r\right)}-p^{\left(r\right)}$ and ${\hat{p}}^{\left(o\right)}-p^{\left(o\right)}$ with $v_{1}/\sqrt{N^{\left(r\right)}}$  and $v_{2}/\sqrt{N}$, respectively. Compute ${\hat{T}}^{b}$ as in (12).

6: **end for**

7: Calculate the p-value:

$$\hat{p}\leftarrow \frac{1}{1+B}(1+\sum_{b=1}^{B}1\{{\hat{T}}^{b}\geq \hat{T}\}).$$

8: **return**$\hat{p}$

### 2.3 Micro-DeMix-2: an EM approach

The proposed Micro-DeMix-1 procedure has strengths in easy and efficient computation: its estimation component can be implemented using standard optimization packages, and its hypothesis-testing component runs quickly due to the use of an asymptotic null distribution. However, it may have a limited scope of applicability due to the numerical evaluation of the log-likelihood in Equation (7). Specifically, based on Equation (2), we know that when ${\left\{\pi_{i}p_{g}^{\left(r\right)}+(1-\pi_{i})p_{g}^{\left(o\right)}\right\}}^{y_{ig}}$ is close to 0, which can occur for rare microbes with extremely small proportions or more common microbes with very large counts, Equation (7) may be evaluated as negative infinity. This happens because, although ${\left\{\pi_{i}p_{g}^{\left(r\right)}+(1-\pi_{i})p_{g}^{\left(o\right)}\right\}}^{y_{ig}}$ is technically not zero, it falls below the smallest representable number in standard software.

To address this issue, instead of working with the marginal density function of $y_{ig}$ in Equation (7), we develop a new estimation procedure based on the joint density function of $y_{ig}$ and $\pi_{i}$, as shown in Equation (2). Specifically, the joint log-likelihood is given by

(15) $$\begin{array}{c} l_{\text{joint}}(\theta ;Y,\pi )=\sum_{i=1}^{S}\;\text{log}\;f(y_{i},\pi_{i}|p^{\left(r\right)},p^{\left(o\right)},a_{1,i},a_{2,i})\\ =\sum_{i=1}^{S}\sum_{g=1}^{G}y_{ig}\;\text{log}(\pi_{i}p_{g}^{\left(r\right)}+(1-\pi_{i})p_{g}^{\left(o\right)})\\ +\sum_{i=1}^{S}\left(a_{1,i}-1)\;\text{log}(\pi_{i}\right)+\sum_{i=1}^{S}\left(a_{2,i}-1)\;\text{log}(1-\pi_{i}\right)\\ -\sum_{i=1}^{S}\;\text{log}\;(B(a_{1,i},a_{2,i}))+C, \end{array}$$

where C is some constant, $Y={\left(y_{1},\ldots ,y_{S}\right)}^{⊺}$, and $\pi ={\left(\pi_{1},\ldots ,\pi_{S}\right)}^{⊺}$. Thanks to the logarithm, Equation (15) is always well defined, regardless of how small ${\left\{\pi_{i}p_{g}^{\left(r\right)}+(1-\pi_{i})p_{g}^{\left(o\right)}\right\}}^{y_{ig}}$ becomes. However, we cannot directly maximize Equation (15) because the $\pi_{i}$ values are not observed. To address this, we develop an EM algorithm (Dempster *et al.* 1977) for estimating θ based on Equation (15). We begin with an initial value $\theta_{0}$. In the kth E-step, we compute $Q_{k}(\theta )=E[l_{\text{joint}}(\theta ,Y,\pi )|\theta_{k-1},y_{i}:i=1,\ldots ,S]$, which is the conditional expectation of $l_{\text{joint}}$ given $y_{i}:i=1,\ldots ,S$ and the values of θ from the previous (k−1)th step for k≥1. Unfortunately, $Q_{k}(\theta )$ does not have a closed form. Thus, we approximate $Q_{k}(\theta )$ using ${\tilde{Q}}_{k}(\theta )=M^{-1}\sum_{m=1}^{M}l_{\text{joint}}(\theta ,Y,{\tilde{\pi }}_{m}^{\left(k\right)})$, where ${\tilde{\pi }}_{m}^{\left(k\right)}$ is a random sample from the conditional distribution of π given Y and $\theta_{k-1}$, generated using the Metropolis–Hastings (MH) algorithm (Hastings 1970). In the kth M-step, we then obtain $\theta_{k}$ by maximizing ${\tilde{Q}}_{k}(\theta )$ with existing optimization tools such as the Nelder–Mead algorithm. The final estimate $\hat{\theta }={\left({\hat{p}}^{\left(r\right)},{\hat{p}}^{\left(o\right)},{\hat{\beta }}_{0},\hat{\beta },{\hat{\gamma }}_{0},\hat{\gamma }\right)}^{⊺}$ is obtained by iterating the EM algorithm between the E-step and M-step until convergence.

Algorithm 3. Estimating $\left\{p_{g}^{\left(o\right)},\beta_{0},\beta ,\gamma_{0},\gamma \right\}$: an EM approach

**Require:**$y_{ig},x_{i}$, an integer K, an integer M, a small number σ (e.g. σ=0.0001)

1: **Initialize:**$\{p_{g}^{\left(o\right)},\beta_{0},\beta ,\gamma_{0},\gamma \}={\left\{p_{g}^{\left(o\right)},\beta_{0},\beta ,\gamma_{0},\gamma \right\}}^{0}$

2: **for**k=1,…,K**do**

3:  (E-step) Generate a random sample ${\tilde{\pi }}_{m}^{\left(k\right)}$ from the  conditional distribution of π given Y and $\theta^{\left(k-1\right)}$ using the Metropolis–Hastings (MH) algorithm. Approximate the conditional expectation $Q_{k}(\theta )=E[l_{\text{joint}}(\theta ,Y,\pi )|\theta^{\left(k-1\right)},y_{i}:i=1,\ldots ,S]$ using ${\tilde{Q}}_{k}(\theta )=M^{-1}\sum_{m=1}^{M}l_{\text{joint}}(\theta ,Y,{\tilde{\pi }}_{m}^{\left(k\right)})$,

4:  (M-step) Obtain $\theta^{\left(k\right)}$ by maximizing the objective  function ${\tilde{Q}}_{k}(\theta )$.

5:  **if**$\sum \left\|\theta^{\left(k\right)}-\theta^{\left(k-1\right)}\right\|\leq \sigma$**then**

6:   **return**$\hat{\theta }={\left\{p_{g}^{\left(o\right)},\beta_{0},\beta ,\gamma_{0},\gamma \right\}}^{k}$

7:  **end if**

8: **end for**

9: **return**$\hat{\theta }={\left\{p_{g}^{\left(o\right)},\beta_{0},\beta ,\gamma_{0},\gamma \right\}}^{K}$

Due to the additional variation introduced by the EM algorithm, Algorithm 2 is no longer suitable for generating p-values for testing $H_{0}:p^{\left(o\right)}=p^{\left(r\right)}$ based on the EM estimates. We fix this issue by developing a permutation-based procedure that works seamlessly with the EM algorithm; see Algorithm 4. Recall that $y_{jg}^{\left(r\right)}$ denotes the absolute abundance for taxon g in the jth subject of the rectum dataset and $x_{j}^{\left(r\right)}$ is the corresponding covariate. Letting $y_{j}^{\left(r\right)}={\left(y_{j1}^{\left(r\right)},\ldots ,y_{jG}^{\left(r\right)}\right)}^{⊺}$, we first randomly select S individuals from $\left\{y_{1}^{\left(r\right)},\ldots ,y_{S_{r}}^{\left(r\right)},y_{1},\ldots ,y_{S}\right\}$ to form a permuted stool microbiome dataset, with the remaining $S_{r}$ subjects forming a permuted rectum microbiome dataset. We also obtain the permuted covariates corresponding to the permuted stool sample. Next, we use the permuted rectum sample to estimate $p^{\left(r\right)}$ and apply the EM algorithm to the permuted stool microbiome data and covariates to obtain an estimate of θ, denoted by ${\hat{\theta }}_{\text{perm}}={\left({\hat{p}}_{\text{perm}}^{\left(r\right)},{\hat{p}}_{\text{perm}}^{\left(o\right)},{\hat{\beta }}_{0,\text{perm}},{\hat{\beta }}_{\text{perm}},{\hat{\gamma }}_{0,\text{perm}},{\hat{\gamma }}_{\text{perm}}\right)}^{⊺}$. We then calculate the permuted test statistic $T_{\text{perm}}=||{\hat{p}}_{\text{perm}}^{\left(r\right)}-{\hat{p}}_{\text{perm}}^{\left(o\right)}|{|}^{2}$. This permutation procedure is repeated B times to generate B permuted test statistics $T_{\text{perm}}^{\left(1\right)},\ldots ,T_{\text{perm}}^{\left(B\right)}$. Finally, the permutation p-value for testing $H_{0}:p^{\left(r\right)}=p^{\left(o\right)}$ is defined as the proportion of $T_{\text{perm}}^{\left(1\right)},\ldots ,T_{\text{perm}}^{\left(B\right)}$ that are larger than or equal to the unpermuted test statistic $T=||{\hat{p}}^{\left(r\right)}-{\hat{p}}^{\left(o\right)}|{|}^{2}$.

Algorithm 4. Permutation method of testing $H_{0}:p^{\left(o\right)}=p^{\left(r\right)}$

**Require:**$y_{jg}^{\left(r\right)},y_{ig},x_{i},x_{j}^{\left(r\right)}$, a large integer B

1: Estimate ${\hat{p}}^{\left(r\right)}$ and ${\hat{p}}^{\left(o\right)}$ using Algorithm 3 in Section 2.3.

2: Compute $\hat{T}$ as in (11) using ${\hat{p}}^{\left(o\right)}$ and ${\hat{p}}^{\left(r\right)}$.

3: **for**b=1,…,B**do**

4:  Randomly permute data ${\left\{(y_{j}^{\left(r\right)},x_{j}^{\left(r\right)})\right\}}_{j=1}^{S^{\left(r\right)}},{\left\{(y_{i},x_{i})\right\}}_{i=1}^{S}$ to  generate permuted rectum data and permuted stool data ${\left\{({\tilde{y}}_{j}^{\left(r\right)},{\tilde{x}}_{j}^{\left(r\right)})\right\}}_{j=1}^{S^{\left(r\right)}},{\left\{({\tilde{y}}_{i},{\tilde{x}}_{i})\right\}}_{i=1}^{S}$.

5:  Estimate ${\hat{p}}_{\text{perm}}^{\left(r\right)}$ and ${\hat{p}}_{\text{perm}}^{\left(o\right)}$ using permutated  data ${\left\{({\tilde{y}}_{j}^{\left(r\right)},{\tilde{x}}_{j}^{\left(r\right)})\right\}}_{j=1}^{S^{\left(r\right)}},{\left\{({\tilde{y}}_{i},{\tilde{x}}_{i})\right\}}_{i=1}^{S}$.

6:  Compute ${\hat{T}}_{\text{perm}}^{\left(b\right)}=||{\hat{p}}_{\text{perm}}^{\left(r\right)}-{\hat{p}}_{\text{perm}}^{\left(o\right)}|{|}^{2}$.

7: **end for**

8: Calculate the p-value:

$$\hat{p}\leftarrow \frac{1}{1+B}(1+\sum_{b=1}^{B}1\{{\hat{T}}_{\text{perm}}^{\left(b\right)}\geq \hat{T}\}).$$

9: **return**$\hat{p}$

Compared to Micro-DeMix-1, the proposed EM algorithm overcomes the numerical issues caused by rare microbes or large counts, giving it broader applicability. However, the EM algorithm is more computationally intensive due to its relatively slow convergence rate and the repeated use of the MH algorithm in the E-step. Additionally, the permutation test requires running the EM algorithm multiple times, making it less efficient than Algorithm 2, which relies on the asymptotic null distribution. Furthermore, as shown in the simulation studies below, Micro-DeMix-1 may have higher power than the EM algorithm in numerical applications.

## 3 Results

We first investigate the finite-sample performance of Micro-DeMix using simulation. We studied the accuracy in the estimation of $p^{\left(o\right)}$ and the type-I error rate and power when testing for differential abundance between the gut microbiome in the rectum and other GI locations.

For rectum samples, we simulated the microbial counts $y_{j}^{\left(r\right)}$ from a $\text{Multinomial}(N_{j}^{\left(r\right)},p^{\left(r\right)})$ distribution with $p_{g}^{\left(r\right)}=1/G$ for g=1,…,G and $j=1,\ldots ,S^{\left(r\right)}$. We next generated the stool microbiome data using the proposed beta-multinomial model. Specifically, we first set $\left(\beta_{0},\beta ,\gamma_{0},\gamma )=(0.1,0.1,0.2,0.1\right)$ and generated the covariate $x_{i}$ from the standard normal distribution. We then randomly generated $\pi_{i}$ from $\text{Beta}(a_{1,i},a_{2,i})$, where $a_{1,i}$ and $a_{2,i}$ are defined in Equations (3)–(6). We assigned $u_{g}$ at evenly spaced values from −1 to 1 for g=1,…,G−1 and let $u_{G}=-\sum_{g=1}^{G-1}u_{g}$. We computed $p_{g}^{\left(o\right)}$ from $u_{g}$ according to Equation (10). Finally, we simulated the stool microbiome data $y_{i}$ from a $\text{Multinomial}(N_{i},p_{i})$ distribution where $p_{i}$ is defined in Equation (1) for i=1,…,S.

### 3.1 Simulation: Estimation

We assessed the performance of Micro-DeMix in estimating $p^{\left(o\right)}$ under six settings, where we considered $S=S^{\left(r\right)}=100$, $N_{j}^{\left(r\right)}=N_{i}=500,1000,5000$ for all i and j, and G=5,10. Given the small scale of this simulation and the efficiency of the Micro-DeMix-1 algorithm, we only implemented the Micro-DeMix-1 algorithm in this section. We reported relative squared errors (RSE) to quantify the discrepancy between our Micro-DeMix estimators ${\hat{\text{p}}}^{\left(o\right)}$ and the true parameters according to

$$\mathrm{RSE}=\frac{\sum_{g}{\left(p_{g}^{\left(o\right)}-{\hat{p}}_{g}^{\left(o\right)}\right)}^{2}}{\sum_{g}{\left(p_{g}^{\left(o\right)}\right)}^{2}}.$$

The mean and standard deviation of RSE under all six settings are presented in Table 1. It was observed that as the library size increases, both the mean and standard deviation of the RSE tend to decrease. This indicates that the accuracy of estimation improves as we increase the library size. Furthermore, it is noticeable that the RSEs for G=10 are greater than those for G=5. This phenomenon occurs because the true proportions become smaller as the total number of taxa increases. Specifically, by design, $p_{g}^{\left(o\right)}=\{0.059,0.116,0.225,0.439,0.161\}$ when G=5, and $p_{g}^{\left(o\right)}=\{0.031,0.039,0.051,0.065,0.083,0.107,0.137,0.177,0.227,0.083\}$ when G=10. This makes estimation more challenging and contributes to a decrease in relative accuracy.

**Table 1.** Mean and standard deviation of RSE obtained under each setting while estimating $p^{\left(o\right)}$ in Micro-DeMix.

	G=5			G=10		
Library size	500	1000	5000	500	1000	5000
Mean	0.00049	0.00035	0.00014	0.00557	0.00501	0.00364
Stand. Dev.	0.00045	0.00035	0.00027	0.00261	0.00201	0.00111

We also examined the performance of Micro-DeMix-EM using a larger-scale simulation study with G=100, as detailed in the Supplementary Material.

### 3.2 Simulation: Type-I error rate and power

In this subsection, we assessed the empirical type-I error rate and power of both Micro-DeMix testing procedures to detect differential abundance between rectal microbes and microbes from other GI locations. Accordingly, we increased the total number of microbes to G=10,20. The data-generating process was the same as that in the previous section except for a few modifications to facilitate the assessment of the type-I error rate and power. Specifically, we set $\left(\beta_{0},\beta ,\gamma_{0},\gamma )=(0.1,0.1,0.7,0.2\right)$ and generated the covariate $x_{i}$ from a normal distribution N(10,0.1). For each G, we generated $p^{\left(r\right)}$ by allowing each $p_{g}^{\left(r\right)}$ and $p_{g}^{\left(o\right)}$ to differ by δ: $p_{g}^{\left(r\right)}=p_{g}^{\left(o\right)}+\delta$ for g=1,…,G/2, and $p_{g}^{\left(r\right)}=p_{g}^{\left(o\right)}-\delta$ for g=G/2+1,…,G. This process ensures that $\sum_{g=1}^{G}p_{g}^{\left(r\right)}=1$ for any δ. We considered relatively weak signals, i.e. δ=0,0.0004,0.0008,…,0.002, for G=10, and further decreased the signal strength by considering δ=0,0.0002,0.0004,…,0.001 for G=20. To ensure a large enough sample size and library size for signal detection, we considered $S=S^{\left(r\right)}=100$, $N_{j}^{\left(r\right)}=N_{i}=30\;000$ for G=10 and $N_{j}^{\left(r\right)}=N_{i}=35\;000$ for G=20.

We compared the performance of our method with seven existing differential abundance tools for microbiome data, including ANCOM-BC (Mandal *et al.* 2015), DESeq2 (Love *et al.* 2014), edgeR (Robinson *et al.* 2010), limma voom (Ritchie *et al.* 2015), MaAsLin2 (Mallick *et al.* 2020), t-test, and Wilcoxon test, using simulated datasets with two sample groups. The Wilcoxon test and t-test were conducted using the R functions wilcox.test and t.test, respectively. ANCOM-BC, which analyzes microbiome compositions, was performed using the ancombc function from the R package ANCOMBC. EdgeR, DESeq2, limma voom, and MaAsLin2 were implemented based on microbial counts using the R packages edgeR, DESeq2, limma, and Maaslin2, respectively.

However, all these existing methods perform separate univariate tests for individual taxa, while our proposed Micro-DeMix focuses on global testing for a group of microbes. To facilitate a meaningful comparison, we applied Bonferroni correction to the univariate p-values generated by each existing method to control the family-level type-I error rate, rejecting the global null hypothesis if at least one adjusted p-value was below the significance level of α=0.05.

We implemented the proposed Micro-DeMix testing procedure (detailed in Algorithm 2) to produce a p-value for testing $H_{0}:p^{\left(r\right)}=p^{\left(o\right)}$ based on 10 000 simulated datasets from the null distribution; i.e. B=10,000 in Algorithm 2. Meanwhile, we also applied the Micro-DeMix-EM testing procedure as outlined in Algorithm 4 to obtain a p-value with B=100. A smaller value of B was chosen for the permutation procedure to reduce the computational burden of Algorithm 4.

**Figure 1.**
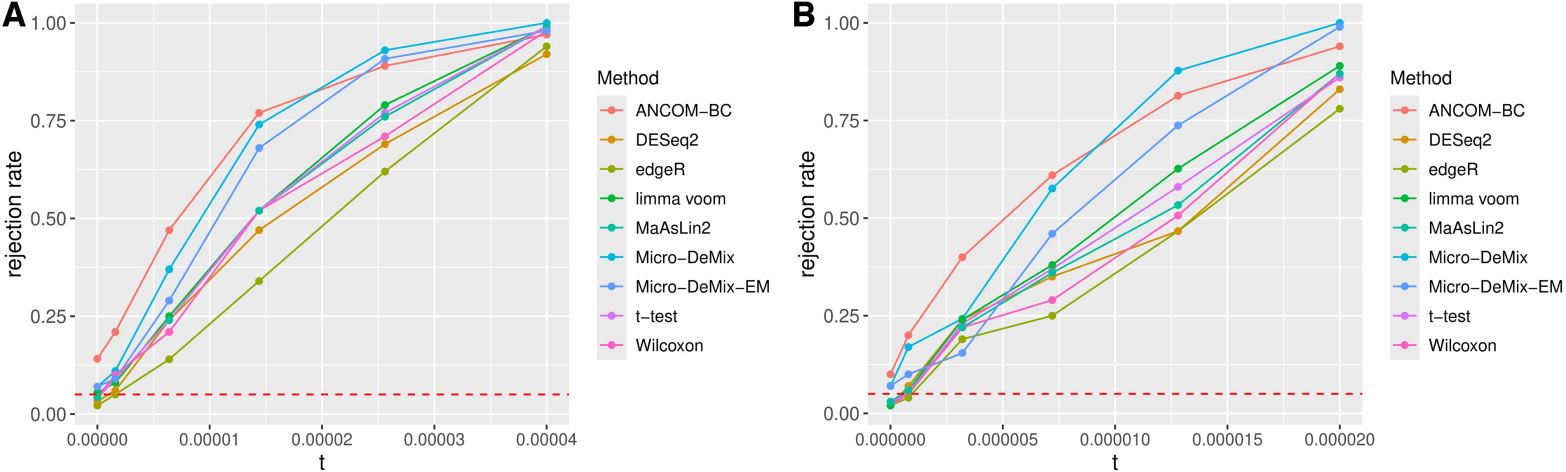
Type-I error rates and powers obtained at various signal strengths from the simulation study. (A) Power curves for *G* = 10. (B) Power curves for *G* = 20. A horizontal dashed line is shown at 0.05; *t* on the *x*-axis denotes the total signal. Rejection rates were obtained using both Micro-DeMix procedures and seven existing tools.

Based on 100 independent replications and the significance level α=0.05, for δ=0 and δ>0, the rejection rate in Fig. 1 represents the type-I error rate and the power, respectively. The value t on *x*-axis represents the total signal, and some calculations yield that $t=||p^{\left(r\right)}-p^{\left(o\right)}|{|}_{2}^{2}=G\delta^{2}$. The points at 0 on *x*-axis representing the scenario that $p^{\left(r\right)}=p^{\left(o\right)}$.

As shown in Fig. 1, the power increases as the total signal grows. Micro-DeMix-EM exhibits lower power than Micro-DeMix, likely due to the slow convergence rate of the EM algorithm and/or the reduced number of permutations. Nonetheless, both Micro-DeMix procedures outperform all existing methods, except for ANCOM-BC in terms of power, but ANCOM-BC exhibits the highest type-I error rate. This result highlights that Micro-DeMix offers the best balance between type-I error rate and power among the methods evaluated. The reason most existing methods lack power is that they overlook the fact that the stool microbiome is a mixed population of rectum microbes and microbes from other GI locations. ANCOM-BC’s distinct performance warrants further benchmarking studies to better understand its unique behavior.

##  

We next revisit the iHMP IBD cohort, which is briefly discussed in Section 1, to demonstrate the effectiveness and efficiency of the proposed Micro-DeMix in elucidating the composition of real fecal microbiome data. We are particularly interested in understanding the heterogeneity of the stool microbiome, how the stool microbiome differs from the rectum microbiome in IBD populations, and how that difference may be related to the pathology of IBD.

As discussed in Section 1, the IBD cohort was a longitudinal study, collecting taxonomic data on the microbiome from 132 individuals (104 IBD patients) by analyzing 1785 stool samples and 651 intestinal biopsies over the course of 1 year (Integrative HMP *et al.* 2019). This IBD cohort was part of the iHMP, which was designed to explore host–microbiome interplay to gain a holistic view of host–microbe interactions (Integrative HMP *et al*. 2019). The IBD IBDMDB research team collected microbiome data from different sites within the human gut of individuals with IBD to understand how the microbiome impacts human health and disease. For a fair comparison, our analysis focused on stool and rectum samples collected at the baseline, resulting in 13 samples for stool and 38 samples for rectum data in IBD patients. After excluding subjects with missing covariates of interest, such as age and gender, the stool group was narrowed down to 12 subjects.

The operational taxonomic unit (OTU)-level data for both groups are highly zero-inflated. More than 90% of the entries in the OTU table derived from the stool samples are zero. Therefore, in the results below, we took a hierarchical approach to investigate the microbial composition of the fecal microbiome as compared to the rectum microbiome at higher taxonomic levels to mitigate the potential influences of excess zeros. We also noticed significant variations in total reads (library size) across samples for both groups, specifically, ranging from 1499 to 79 228 for stool samples and from 34 to 31 781 for rectum samples. This nonbiological variation can result in samples with disproportionately high read counts dominating the analysis under the multinomial model, potentially masking true biological patterns due to this technical factor. We mitigated this issue by performing rarefaction, using the R function rarefy_even_depth from package phyloseq as in the exiting literature (McMurdie and Holmes 2013). In microbial studies, such rarefying has been used for normalization by randomly subsampling the data to mitigate the potential influence of varying library sizes (Hughes and Hellmann 2005, Koren *et al.* 2013, Navas-Molina *et al.* 2013). We also acknowledge that there is an ongoing debate in the research community regarding the use of rarefaction; see McMurdie and Holmes (2014) for more details. Given this, we suggest that the decision to perform rarefaction in microbiome data analysis should be carefully considered based on the data characteristics, research goals, and biological interpretations. We also applied the Micro-DeMix-EM algorithm to the IBD data without performing rarefaction, and the results are presented in the Supplementary Material.

### 3.3 iHMP IBD data: Phylum-level analysis

We aggregated the absolute counts from the OTU level to the phylum level, resulting in eight phyla, leading to a much lower percentage of zeros compared to the OTU-level data (50.9% versus 93.6%). We used 1499 as the rarefaction level for stool samples, as it is the minimum library size in the stool group. For a fair comparison, we applied the same rarefaction level to rectum samples, resulting in 30 samples in rectum group. Since this procedure is based on random subsampling without replacement, we repeated the procedure 100 times and took the average to reduce variability for both groups. Samples with library sizes less than 1499 were removed from the analysis.

**Figure 2.**
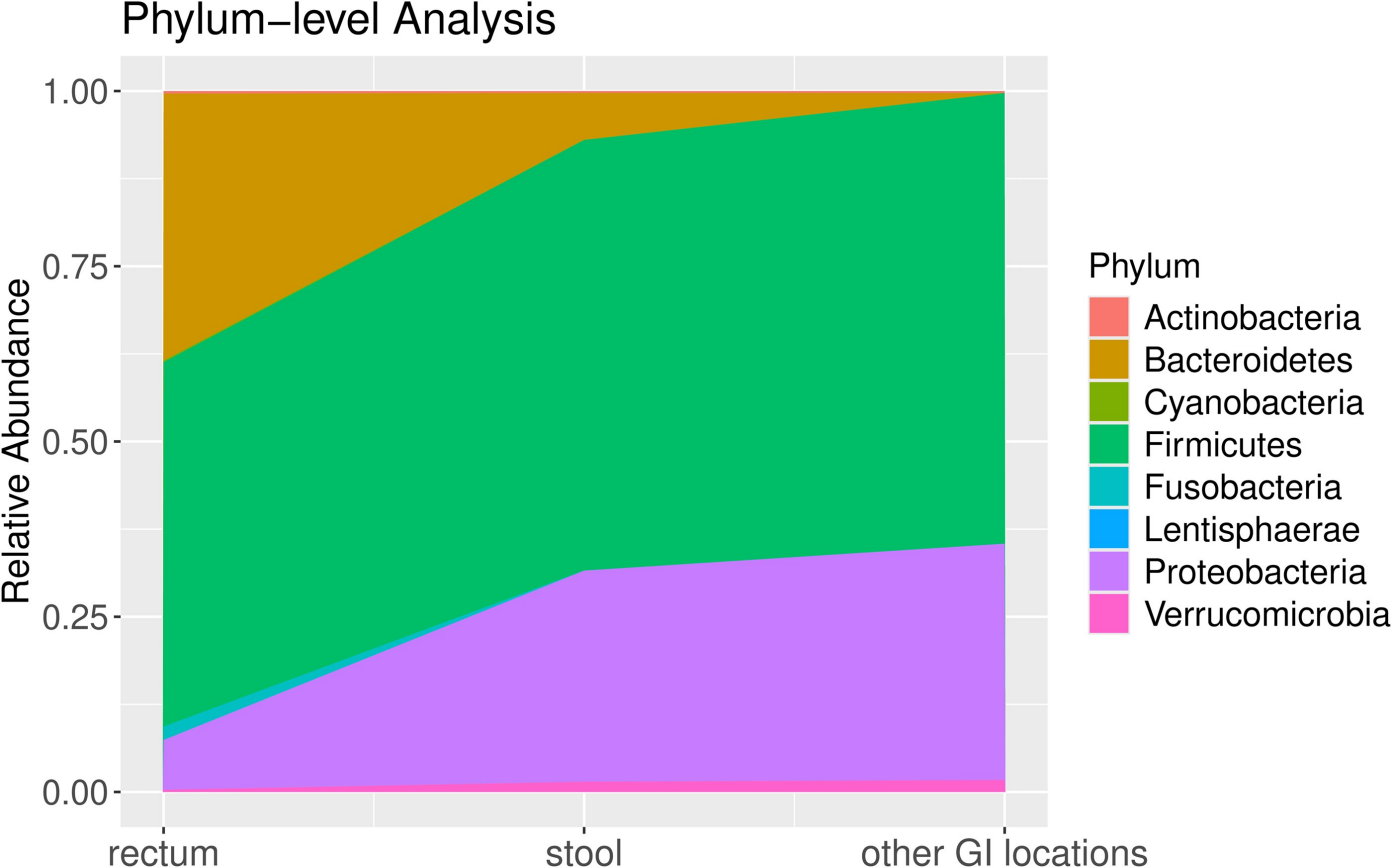
Relative abundance of microbial populations at the phylum level in the rectum, stool, and other GI locations.

We computed the relative abundance of these eight phyla in both the rectum and stool samples, and the results revealed a clear difference in the microbiome composition (Fig. 2).

Phylum Firmicutes was highly abundant in both the rectum and stool. It was the major phylum detected, representing more than 50% of the total microbial relative abundance in both groups. Overall, the relative abundance of Firmicutes in the stool (∼61.4%) was slightly higher than that in the rectum (∼52.0%). When compared to the microbial profile in rectum, the stool samples exhibited a considerable increase in the relative abundance of phylum Proteobacteria (from 7.1% to 30.1%), while the relative abundance of Bacteroidetes decreased from 38.1% to 6.7%. In addition, we observed an elevated relative abundance of the Verrucomicrobia phylum in the stool microbiome compared to the rectum, with an increase from 0.31% to 1.5%. Conversely, the phylum Fusobacteria showed a decrease from over 1.9% to a lower level. The remaining three phyla were nearly imperceptible in rectum and stool.

To further understand the composition of the fecal microbiome in IBD populations, we fitted the proposed Micro-DeMix model, incorporating gender and age as covariates. This analysis revealed estimates of the relative abundance of selected phyla from the other GI locations (Fig. 2). Compared to the rectum microbiome, the microbial profile of other GI locations revealed a higher relative abundance of phylum Firmicutes (∼64.3%), Proteobacteria (∼33.7%), and Verrucomicrobia (∼1.7%). Phylum Bacteroidetes was undetectable in other GI locations. The remaining phyla also exhibited relatively low abundance (<1%). Based on 10 000 simulated datasets, the Micro-DeMix test suggested significant differences in the taxonomic composition of the eight phyla between the rectum and other GI locations in IBD populations (*p*-value <1e−4).

Our findings are consistent with recent studies on the gut microbiome in IBD populations. Specifically, the rectum and fecal microbiome of IBD patients is dominated by three major bacterial phyla: Firmicutes, Bacteroidetes, Proteobacteria, and to a lesser degree Verrucomicrobia and Actinobacteria (Frank *et al.* 2007, Lo Presti *et al.* 2019). Our results are also consistent with recent studies that described distinct changes in the composition of the microbiota along the GI tract in IBD populations. The lower GI tract exhibited a larger abundance of Firmicutes and Bacteroidetes, whereas the upper GI tract was predominated by Proteobacteria and Firmicutes (Vuik *et al.* 2019). Compared to healthy individuals, the reduction in the relative abundance of Firmicutes and an elevation in Bacteroidetes have been observed in IBD patients (Khan *et al.* 2019, Lo Presti *et al.* 2019, Vuik *et al.* 2019, Zhang *et al.* 2022). Thus, our results shed light on the scientific premise of developing an effective microbiome-based treatment approach for IBD, such as the therapeutic supplementation of probiotics, prebiotics, and symbiotics, and fecal microbiota transplantation.

### 3.4 iHMP IBD data: Microbial composition within Proteobacteria and Firmicutes

**Figure 3.**
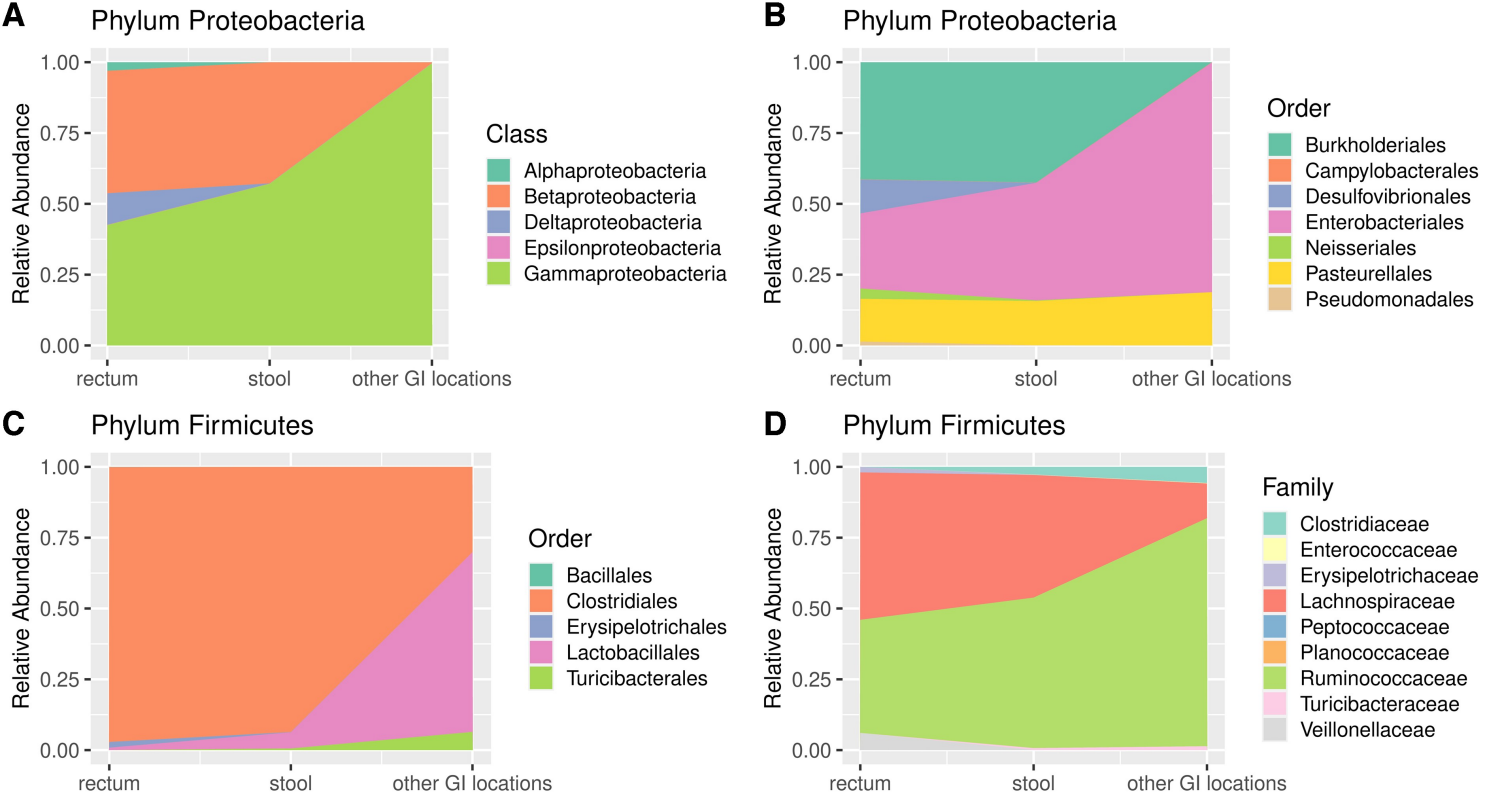
Relative abundance of microbial populations at various taxonomic levels in the rectum, stool, and other GI locations. (A, B) Microbial composition at the class and order levels within the phylum Proteobacteria. (C, D) Microbial composition at the order and family levels within the phylum Firmicutes.

In Section 4.1, we identified Proteobacteria and Firmicutes as the most abundant phyla in stool samples. To further understand the microbes in these two phyla, we conducted analyses of microbial composition at lower taxonomic levels (class, order, family) within Proteobacteria and Firmicutes phylum (Fig. 3).

Similar to the analyses in Section 4.1, we computed the relative abundance of all microbial classes and orders within phylum Proteobacteria for both the stool and rectum samples, and implemented the Micro-DeMix model to elucidate the difference between the rectum microbiome and the microbes in other GI locations. We followed the rarefying procedure that described in Section 4.1 to process the stool OTU data. In stool samples, the minimum library size is 13, which is not big enough to be included in the study. Therefore, we used 369, the second minimum, as the rarefaction level for both stool and rectum group. The outcomes of our analyses are visually represented in Fig. 3A and B.

Fig. 3A reveals that within phylum Proteobacteria, the major classes in rectum and stool are Betaproteobacteria and Gammaproteobacteria. However, the rectum microbiome is distinguished by a higher relative abundance of class Deltaproteobacteria (∼11.1%) and Alphaproteobacteria (∼3.0%), which are significantly low in abundance in stool samples. There is a low relative abundance of Epsilonproteobacteria (<1%) in both stool and rectum. As seen in Fig 3A, Gammaproteobacteria (∼99.6%) is the most dominant class among the selected classes in other GI locations, while the other four classes are nearly undetectable. Although this finding may appear extreme, it is aligned with the existing knowledge that IBD patients have an altered gut microbiota marked by an increased relative abundance of Gammaproteobacteria class (Scales *et al.* 2016).

We proceeded to analyze the microbial composition of the phylum Proteobacteria at the order level, revealing marked differences between the rectum and stool samples (Fig. 3B). While both showed high abundance in orders Burkholderiales, Enterobacteriales, and Pasteurellales, the rectum samples were mainly characterized by the orders Desulfovibrionales and Neisseriales. We utilized the Micro-DeMix method to assess the microbiome composition in other GI locations. Our analysis revealed a dominance of Enterobacteriales (∼81.1%), along with a lower proportion of Pasteurellales (∼18.83%). This finding is supported by a recent study showing that Enterobacteriaceae exhibit increased abundance in the low-pH GI location and ileum (Morgan *et al.* 2012).

The Micro-DeMix test indicates significant differences in microbial profiles between the rectum and other GI locations (*p*-value <1e−4) within the phylum Proteobacteria at both the class and order levels.

We further explored the microbial composition of the Firmicutes phylum. In the stool samples, three classes were identified: Bacilli, Clostridia, and Erysipelotrichi. At the original OTU level, class Clostridia dominated with a percentage of 97.1%, while class Erysipelotrichi was nearly undetectable. Given these findings, we narrowed our focus to lower taxonomic levels, specifically, order and family within the Firmicutes phylum. Similarly, we employed the minimum library size, 493, as the rarefaction level and processed both stool and rectum OTU data. The corresponding results are shown in Fig. 3C and D.

Notably, at the order level (Fig. 3C), the phylum Firmicutes in the rectum and stool was predominantly represented by the order Clostridiales (97.1% in rectum and 93.6% in stool of all Firmicutes). Our results demonstrate the presence of order Turicibacterales in the stool samples, while it is absent in the rectum samples, which suggest that order Turicibacterales in stool samples may originate from other GI locations. The relative abundance of the order Lactobacillales increased in stool compared to rectum from 0.86% to 5.7%. In contrast, order Erysipelotrichales is found to be present (2.0%) in the rectum but remains at lower levels in the stool. The results of Micro-DeMix analysis show a *p*-value <1e−4 for testing differential abundance between the rectum and other GI locations. Meanwhile, it highlights a significantly higher relative abundance of Lactobacillales (∼63.4%) in other GI locations compared to the rectum and stool. The analysis estimates that order Turicibacterales (∼6.4%) is also more abundant in other GI locations. These findings indicate a distinct microbial composition in other GI locations, with increased prevalence of orders Lactobacillales and Turicibacterales.

At the family level within the Firmicutes phylum (Fig. 3D), major families of the rectum microbiota are Ruminococcaceae, Lachnospiraceae, and Veillonellaceae. Comparisons between stool and rectum samples reveal an increase in Ruminococcaceae and a decrease in Lachnospiraceae and Veillonellaceae, aligning with findings from other studies (Lo Presti *et al.* 2019). One significant finding is the presence of the Clostridiaceae, Enterococcaceae, Planococcaceae, and Turicibacteraceae families in stool samples but not in the rectum. This observation suggests that these families detected in stool originate from other GI microbiome community. In other GI locations, our analysis shows a higher abundance of the Clostridiaceae and Ruminococcaceae families. In contrast, the family Lachnospiraceae exhibits a comparatively lower abundance. Furthermore, the *p*-value obtained through Micro-DeMix hypothesis testing is low (<1e−4). These findings imply distinctive microbial profiles among the rectum, stool, and other GI locations, indicating variations in the relative abundance of specific taxa.

## 4 Conclusion

In this paper, we introduced Micro-DeMix, a beta-multinomial model designed for deconvoluting microbial abundance in stool samples. We proposed two parameter estimation procedures for the model, each with its strengths in specific types of applications. Additionally, we developed hypothesis testing procedures for detecting differential abundance, which has proven more effective compared to existing methods. We applied Micro-DeMix to rectum and stool microbiome datasets from the iHMP IBD cohort in elucidating the composition of real fecal microbiome data in IBD populations.

In the analyses of the iHMP IBD data, we performed rarefying on stool and rectum datasets to address the large variations in library sizes across samples. While rarefaction has been widely adopted in real microbiome applications, a limitation of this approach is that the selection of rarefaction level is data-dependent, and different rarefaction levels may lead to slightly different results. Due to the limited sample size, our strategy for selecting the rarefaction level is to keep as many samples as possible while removing samples with unreliably low library sizes. Different strategies may be considered for other datasets.

As mentioned earlier, the current paper focuses on integrating rectum samples and fecal samples due to data availability and biological relevance. However, the proposed method can seamlessly be used for integration of fecal samples with microbiome data collected from other GI locations. Furthermore, with microbiome data available from two or more GI locations, we could extend Micro-DeMix to have a refined decomposition of the fecal microbiome. Specifically, following work could consider the stool microbiome as a *J*-component mixture of gut microbes, i.e. we let $p_{ig}=\pi_{i}^{\left(1\right)}p_{g}^{\left(1\right)}+\cdots +\pi_{i}^{\left(J-1\right)}p_{g}^{\left(J-1\right)}+(1-\pi_{i}^{\left(1\right)}-\cdots -\pi_{i}^{\left(J-1\right)})p_{g}^{\left(J\right)}$ in Equation (1), where $\pi_{i}$ follows a Dirichlet $\left(a_{i}\right)$ distribution. We leave this fruitful area for future research.

## Supplementary Material

btae667_Supplementary_Data

## Data Availability

The IBD data analyzed in the paper can be accessed via HMP portal at https://portal.hmpdacc.org/. Code for implementing our methods is available in the R package MicroDemix, available at https://github.com/liuruoqian/MicroDemix.
