# Supplementary material for: Micro-DeMix: a mixture beta-multinomial model for investigating the heterogeneity of the stool microbiome compositions
Source: Bioinformatics. 2024 Nov 19;40(12):btae667. doi: 10.1093/bioinformatics/btae667 (PMC11645251; doi:10.1093/bioinformatics/btae667)
Supplement: btae667_Supplementary_Data [file btae667_supplementary_data.pdf]

Supplementary to "Micro-DeMix: A mixture beta-multinomial model for investigating the heterogeneity of the stool microbiome compositions"

Ruoqian Liu[1], **Yue Wang**[2], and **Dan Cheng**[1]

[1]*School of Mathematical and Statistical Sciences, Arizona State University*
[2]*Department of Biostatistics and Informatics, Colorado School of Public Health*

November 7, 2024

In this document, Section 1 provides derivations for the mean and variance of y_{ig} under the proposed beta-multinomial model. Section 2 provides the proof of Proposition 1 in the main paper. Section 3 demonstrates the feasibility of the Micro-DeMix-EM algorithm for larger-scale applications with a simulation study with $G = 100$ microbes. In Section 4, we apply the proposed Micro-DeMix-EM algorithm to the IBD data set without performing rarefaction. In Section 5, we repeat the simulation study from Section 3.2 in the main paper, using the Benjamini-Hochberg correction to control the false discovery rate (FDR) for the univariate p-values generated by the existing methods.

1 Mean and Variance of y_{ig}

We first derive the mean and variance of y_{ig} under the proposed beta-multinomial model, as described in Remark 1 in the main paper.

$$
\begin{aligned}
\mathbb{E}(y_{ig}) &= \mathbb{E}(\mathbb{E}(y_{ig}|\pi_i)) \\
&= \mathbb{E}(N_i(\pi_i p_g^{(r)} + (1-\pi_i)p_g^{(o)})) \\
&= N_i[p_g^{(r)}\mathbb{E}(\pi_i) + p_g^{(o)}(1-\mathbb{E}(\pi_i))] \\
&= N_i[p_g^{(r)}\frac{a_{1,i}}{a_{1,i}+a_{2,i}} + p_g^{(o)}(1-\frac{a_{1,i}}{a_{1,i}+a_{2,i}})] \\
\text{Var}(y_{ig}) &= \mathbb{E}(\text{Var}(y_{ig}|\pi_i)) + \text{Var}(\mathbb{E}(y_{ig}|\pi_i)) \\
&= \mathbb{E}[N_i(\pi_i p_g^{(r)} + (1-\pi_i)p_g^{(o)})(1-\pi_i p_g^{(r)} - (1-\pi_i)p_g^{o})] \\
&+ \text{Var}(N_i(\pi_i p_g^{(r)} + (1-\pi_i)p_g^{(o)})) \\
&= N_i\{\mathbb{E}(\pi_i)(p_g^{(r)} - 2p_g^{(r)}p_g^{(o)} - p_g^{(o)} + 2(p_g^{(o)})^2) \\
&+ \mathbb{E}(\pi_i^2)(-(p_g^{(r)})^2 + 2p_g^{(r)}p_g^{(o)} - (p_g^{(o)})^2) + p_g^{(o)} - (p_g^{(o)})^2\} \\
&+ N_i^2(p_g^{(r)} - p_g^{(o)})^2\text{Var}(\pi_i) \\
&= N_i\{\frac{a_{1,i}}{a_{1,i}+a_{2,i}}(p_g^{(r)} - 2p_g^{(r)}p_g^{(o)} - p_g^{(o)} + 2(p_g^{(o)})^2) \\
&+ (\frac{a_{1,i}^2}{(a_{1,i}+a_{2,i})^2} + \frac{a_{1,i}a_{2,i}}{(a_{1,i}+a_{2,i})^2(a_{1,i}+a_{2,i}+1)}) \\
&(-(p_g^{(r)})^2 + 2p_g^{(r)}p_g^{(o)} - (p_g^{(o)})^2) + p_g^{(o)} - (p_g^{(o)})^2\} \\
&+ N_i^2(p_g^{(r)} - p_g^{(o)})^2\frac{a_{1,i}a_{2,i}}{(a_{1,i}+a_{2,i})^2(a_{1,i}+a_{2,i}+1)}
\end{aligned}
$$

2 Proof of Proposition 1

Since $N^{(r)}/N \to K > 0$, and noting that $\mathbf{y}_i$ and $\mathbf{y}_j^{(r)}$ are independent, by the central limit theorem, we have

$$\sqrt{N}\begin{pmatrix}\hat{\boldsymbol{p}}^{(o)}-\boldsymbol{p}^{(o)}\\ \sqrt{K}(\hat{\boldsymbol{p}}^{(r)}-\boldsymbol{p}^{(r)})\end{pmatrix}\xrightarrow{d} N_{2G}\left(\begin{pmatrix}\mathbf{0}\\ \mathbf{0}\end{pmatrix},\begin{pmatrix}V(\boldsymbol{p}^{(r)}) & \mathbf{0}\\ \mathbf{0} & V(\boldsymbol{p}^{(r)})\end{pmatrix}\right). \tag{1}$$

Let $\boldsymbol{x}_1 = \hat{\boldsymbol{p}}^{(o)} - \boldsymbol{p}^{(o)}$, $\boldsymbol{x}_2 = \sqrt{K}(\hat{\boldsymbol{p}}^{(r)} - \boldsymbol{p}^{(r)})$, and let $(\boldsymbol{y}_1, \boldsymbol{y}_2)$ be a random vector with the multivariate normal distribution in (1). Then we can write (1) as

$$\sqrt{N}\begin{pmatrix}\boldsymbol{x}_1\\ \boldsymbol{x}_2\end{pmatrix}\xrightarrow{d}\begin{pmatrix}\boldsymbol{y}_1\\ \boldsymbol{y}_2\end{pmatrix}.$$

Define a continuous function $g(\boldsymbol{y}_1, \boldsymbol{y}_2) = \boldsymbol{y}_1^\intercal\boldsymbol{y}_1 + (1/K)\boldsymbol{y}_2^\intercal\boldsymbol{y}_2 - (2/\sqrt{K})\boldsymbol{y}_1^\intercal\boldsymbol{y}_2$. It is seen that $N\hat{T} = g(\sqrt{N}\boldsymbol{x}_1, \sqrt{N}\boldsymbol{x}_2)$. By the continuous mapping theorem, we obtain $N\hat{T} \xrightarrow{d} g(\boldsymbol{y}_1, \boldsymbol{y}_2)$.

3 Simulation Study with $G = 100$

To demonstrate the estimation accuracy of the Micro-DeMix-EM for larger-scale applications, we conducted a simulation study with $G = 100$ microbes, a realistic number for real 16S applications when focusing on lower taxonomic levels after removing microbes with extremely high zero proportions.

For rectum samples, we simulated the microbial counts $\mathbf{y}_j^{(r)}$ from a Multinomial$(N_j^{(r)}, \boldsymbol{p}^{(r)})$ distribution with $p_g^{(r)} = 0.01$ for $g = 1, ...100$ and $j = 1, \ldots, 100$. We next generated the stool microbiome data using the proposed beta-multinomial model with $(\beta_0, \beta, \gamma_0, \gamma) = (0.1, 0.1, 0.7, 0.2)$,

where x_i were drawn from a normal distribution $N(10, 0.1)$. We then randomly generated π_i from Beta$(a_{1,i}, a_{2,i})$, with $a_{1,i}$ and $a_{2,i}$ defined as

$$\mu_i = \frac{a_{1,i}}{a_{1,i} + a_{2,i}} \tag{2}$$

$$\phi_i = a_{1,i} + a_{2,i} \tag{3}$$

$$\log(\frac{\mu_i}{1-\mu_i}) = \beta_0 + \mathbf{x}_i^\intercal \boldsymbol{\beta}, \tag{4}$$

$$\log(\phi_i) = \gamma_0 + \mathbf{x}_i^\intercal \boldsymbol{\gamma} \tag{5}$$

The $p_g^{(o)}$ were set as follows: $p_g^{(o)} = 0.006$ for $g = 1, \dots, 25$, $p_g^{(o)} = 0.008$ for $g = 26, \dots, 50$, $p_g^{(o)} = 0.012$ for $g = 51, \dots, 75$, and $p_g^{(o)} = 0.014$ for $g = 76, \dots, 100$. Finally, we simulated the stool microbiome data $\mathbf{y}_i$ from a Multinomial$(N_i, \boldsymbol{p}_i)$ distribution where $\boldsymbol{p}_i$ is defined as

$$p_{ig} = \pi_i p_g^{(r)} + (1 - \pi_i) p_g^{(o)} \tag{6}$$

We considered $N_j^{(r)} = N_i = 30000$ for all i and j. We reported relative squared errors (RSE) to quantify the discrepancy between our Micro-DeMix estimators $\hat{\boldsymbol{p}}^{(o)}$ and the true parameters according to

$$RSE = \frac{\sum_g (p_g^{(o)} - \hat{p}_g^{(o)})^2}{\sum_g (p_g^{(o)})^2}.$$

The mean of the RSE is 0.031, with a standard deviation of 0.0007. While the RSE is higher than that reported for $G = 10$ and $G = 20$ due to the larger number of microbes, it still demonstrates Micro-DeMix-EM's capacity for accurately deconvoluting the abundance of a greater number of microbes in real-world applications.

4 Application to the iHMP IBD Data

In this section, we analyze the iHMP IBD dataset, as described in Section 4 of the main manuscript, using the proposed Micro-DeMix-EM algorithm without applying rarefaction. Our objective is to demonstrate the potential impact of rarefaction in this context, where the library sizes vary significantly across individuals.

4.1 Phylum-level Analysis

We aggregated the absolute counts from the OTU level to the phylum level, resulting in 8 phyla and a significantly lower percentage of zeros compared to the OTU-level data (50.9% vs. 93.6%). We computed the relative abundance of these 8 phyla in both the rectum and stool samples, and the results revealed a clear difference in the microbiome composition (Fig. 1). Phylum

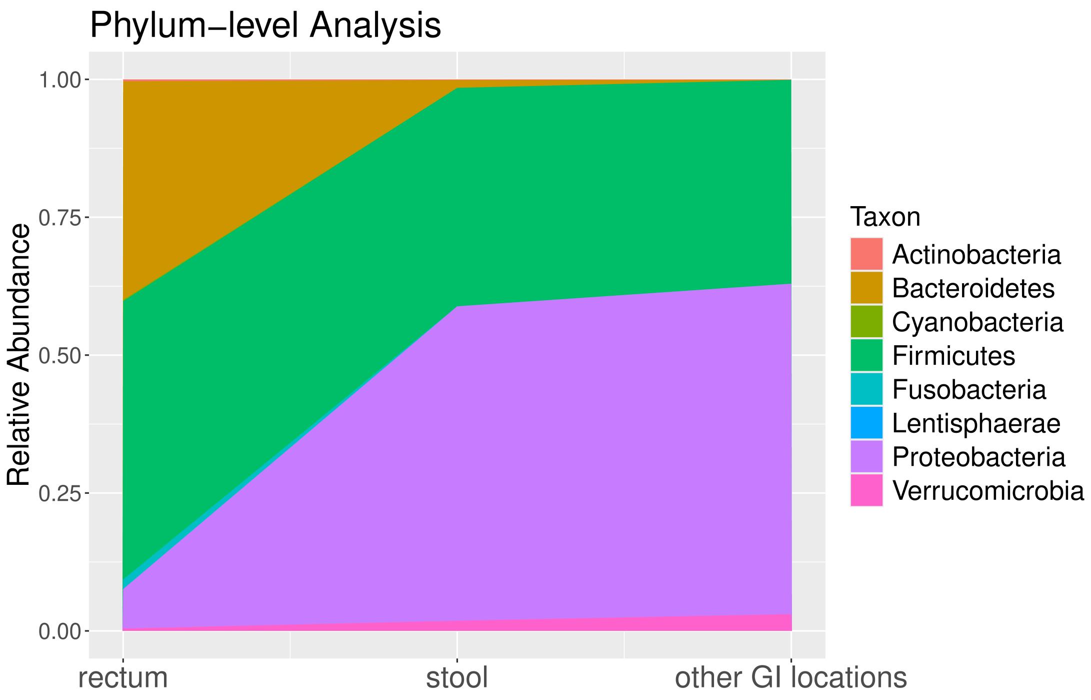

Figure 1: Relative abundance of microbial populations at the phylum level in the rectum, stool, and other GI locations.

Firmicutes was highly abundant in both the rectum and stool. Overall, the relative abundance

of Firmicutes in the rectum ($\sim$ 50.5%) was slightly higher than that in the stool ($\sim$ 39.6%). When compared to the microbial profile in rectum, the stool samples exhibited a considerable increase in the relative abundance of phylum Proteobacteria (from 7.2% to 57.0%), while the relative abundance of Bacteroidetes decreased from 39.7% to 1.4%. In addition, we observed an elevated relative abundance of the Verrucomicrobia phylum in the stool microbiome compared to the rectum, with an increase from 0.43% to 1.8%. Conversely, the phylum Fusobacteria showed a decrease from over 1.7% to a lower level ($<$ 0.1%). The remaining three phyla were nearly imperceptible ($<$ 1%) in rectum and stool.

To further understand the composition of the fecal microbiome in IBD populations, we fitted the proposed Micro-DeMix model using the EM algorithm, incorporating gender and age as covariates. This analysis revealed estimates of the relative abundance of selected phyla from the other GI locations (Figure 1). Compared to the rectum microbiome, the microbial profile of other GI locations revealed a lower relative abundance of phylum Firmicutes ($\sim$ 37.0%), and higher in Proteobacteria ($\sim$ 59.9%) and Verrucomicrobia ($\sim$ 3.0%). Actinobacteria exhibited relatively low abundance ($<$ 0.1%). Phylum Bacteroidetes, Cyanobacteria, Fusobacteria, and Lentisphaerae were not observed in other GI locations. Based on the permutation testing procedure as detailed in Algorithm 4, the test suggested insignificant differences in the taxonomic composition of the 8 phyla between the rectum and other GI locations in IBD populations with p-value = 0.3.

4.2 Microbial Composition within Proteobacteria and Firmicutes

In Section 4.1, we identified Proteobacteria and Firmicutes as the abundant phyla in stool samples. To further understand the microbes in these two phyla, we conducted analyses of microbial composition at lower taxonomic levels (class, order, family) within Proteobacteria and Firmicutes phylum (Fig 2).

Similar to the analyses in Section 4.1, we computed the relative abundance of all microbial

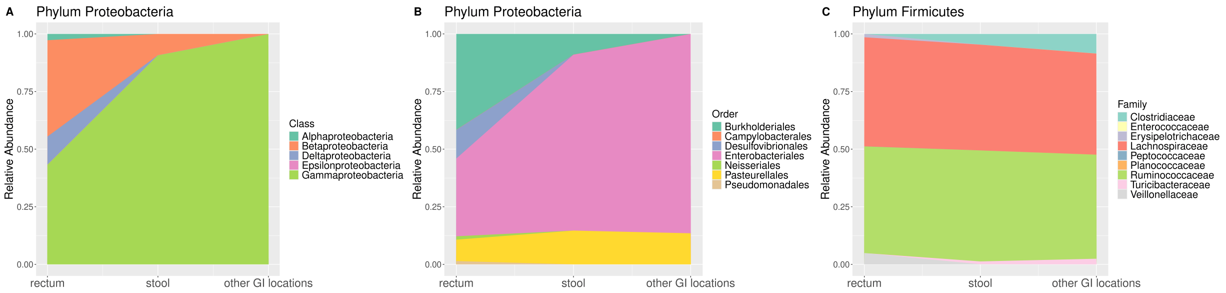

Figure 2: Relative abundance of microbial populations at various taxonomic levels in the rectum, stool, and other GI locations. Plot A and B demonstrate the microbial composition at the class and order levels within the phylum Proteobacteria. Plot C demonstrate the microbial composition at the family levels within the phylum Firmicutes.

classes and orders within phylum Proteobacteria for both the stool and rectum samples, and implemented the Micro-DeMix model to elucidate the difference between the rectum microbiome and the microbes in other GI locations. The outcomes of our analyses are visually represented in Figure 2A and Figure 2B.

Figure 2A reveals that within phylum Proteobacteria, the major classes in rectum and stool are Betaproteobacteria and Gammaproteobacteria. However, the rectum microbiome is distinguished by a higher relative abundance of class Deltaproteobacteria ($\sim 12.1\%$) and Alphaproteobacteria ($\sim 2.7\%$), which are significantly low in abundance in stool samples. There is a low relative abundance of Epsilonproteobacteria ($< 0.1\%$) in both stool and rectum. As seen in Fig 2A, Gammaproteobacteria ($\sim 99.9\%$) is the most dominant class among the selected classes in other GI locations, while the other 4 classes are nearly undetectable.

We proceeded to analyze the microbial composition of the phylum Proteobacteria at the order level, revealing marked differences between the rectum and stool samples (Figure 2B). While both showed high abundance in orders Burkholderiales, Enterobacteriales and Pasteurellales, the rectum samples was mainly charaterized by the orders Desulfovibrionales, Neisseriales and Pseudomonadales. We utilized the Micro-DeMix method to assess the microbiome composition in other GI locations. Our analysis revealed a dominance of Enterobacteriales ($\sim 86.5\%$), along with a lower proportion of Pasteurellales ($\sim 13.5\%$). It is worth noticing that order Burkholde-

riales, Desulfovibrionales, Neisseriales and Pseudomonadales are absent in other GI locations, while Campylobacterales is low abundance ($< 0.1\%$) in all rectum, stool and other GI locations.

The Micro-Demix test indicates no significant differences in microbial profiles between the rectum and other GI locations within the phylum Proteobacteria with a p-value of 0.53 at the class level and a p-value of 0.48 at the order level.

We further explored the microbial composition of the Firmicutes phylum. In the stool samples, three classes were identified: Bacilli, Clostridia, and Erysipelotrichi. At the original OTU-level, class Clostridia dominated with a percentage of 97.1%, while class Erysipelotrichi was nearly undetectable. Similarly, within the phylum Firmicutes, the order Clostridiales accounted for 97.1%, followed by Lactobacillales at 1.8% and Turicibacterales at 1.1%. Given these findings, we narrowed our focus to lower taxonomic levels, specifically, family within the Firmicutes phylum. The corresponding results are shown in Figure 2C.

At the family level within the Firmicutes phylum (Figure 2C), major families of the rectum microbiota are Lachnospiraceae ($\sim$ 47.3%), Ruminococcaceae ($\sim$ 46.3%), and Veillonellaceae ($\sim$ 4.9%). Comparisons between stool and rectum samples reveal an increase in Ruminococcaceae ($\sim$ 48.1%) and a decrease in Lachnospiraceae ($\sim$ 45.9%) and Veillonellaceae ($< 0.1\%$). One significant finding is the presence of the Clostridiaceae, Enterococcaceae, Planococcaceae, and Turicibacteraceae families in stool samples but not in the rectum. This observation suggests that these families detected in stool originate from other gastrointestinal (GI) microbiome community. In other GI locations, our analysis shows a higher abundance of the Clostridiaceae ($\sim$ 8.5%) and Turicibacteraceae ($\sim$ 2.5%) families. In contrast, the family Lachnospiraceae and Ruminococcaceae exhibits a comparatively lower abundance. Furthermore, the p-value obtained through Micro-Demix hypothesis testing is 0.7, indicating no significant variation in microbial compositions among rectum and other GI locations.

4.3 Comparison with the results in the main manuscript

Several major differences are noted between the results in Sections 4.1 and 4.2 and those in the main manuscript where rarefaction is performed.

At the phylum level in stool microbiome, not performing rarefaction leads to an increased relative abundance of Proteobacteria and a decreased relative abundance of Bacteroidetes and Firmicutes. This thus affects the estimated microbial composition in other gastrointestinal (GI) locations. Specifically, the estimated relative abundance of Firmicutes decreases from 64.3% to 37.0%, while Proteobacteria increases from 33.7% to 59.9% after rarefaction is excluded.

At the class level within Proteobacteria, Gammaproteobacteria exhibit a higher relative abundance in stool samples without rarefaction. Similarly, at the order level within Proteobacteria, Enterobacteriales show increased dominance in stool, a pattern not observed in previous analyses with rarefaction. Despite these changes, the estimated microbial composition in other GI locations obtained through deconvolution was not significantly affected.

Within the phylum Firmicutes, the order Clostridiales becomes more dominant (over 95%) across all three locations - rectum, stool, and other GI sites - when rarefaction is excluded. Additionally, the family Lachnospiraceae shows an increased presence in stool samples without rarefaction. After applying Micro-DeMix, the microbial composition in other GI locations is characterized by a significant increase in Lachnospiraceae and a decrease in Ruminococcaceae compared to results obtained with rarefaction.

None of the Micro-DeMix-EM p-values yield significant differences, while all comparisons in the main manuscript yield significant differences. This may be due to the relatively lower power of the EM method, as observed in the simulation studies.

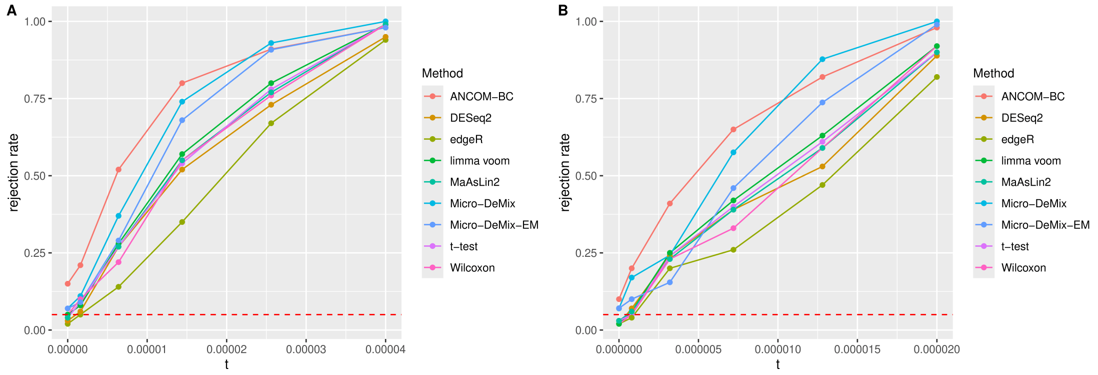

Figure 3: Type I error rates and statistical power at varying signal strengths from the simulation study with Benjamini-Hochberg correction. Plot A corresponds to $G = 10$, and plot B to $G = 20$. A horizontal dashed line indicates the 0.05 significance threshold. The x-axis (t) represents the total signal.

5 Simulation Study: Benjamini-Hochberg Correction

To evaluate the impact of different multiple testing correction methods on statistical power, we repeated the simulation study from Section 3.2 of the main paper, applying the Benjamini-Hochberg (BH) correction (Benjamini and Hochberg, 1995) to control the false discovery rate (FDR) for the univariate p-values generated by the existing methods. These methods include ANCOM-BC (Mandal et al., 2015), DESeq2 (Love et al., 2014), edgeR (Robinson et al., 2010), limma voom (Ritchie et al., 2015), MaAsLin2 (Mallick et al., 2020), the t-test, and the Wilcoxon test. As in the original analysis, we rejected the global null hypothesis if at least one adjusted p-value was below the significance level of $\alpha = 0.05$. We present the results for Benjamini-Hochberg correction in Fig. 3.

Compared to the Bonferroni-corrected results in Fig. 1 of the main paper, the new results in Fig. 3 are consistent across both $G = 10$ and $G = 20$. Our Micro-DeMix procedures outperformed all existing methods in terms of power, with the exception of ANCOM-BC, which exhibited the highest type-I error rate. As expected, the power of the existing methods improved under the BH correction compared to the Bonferroni correction, reflecting the BH procedure's less conservative nature, but the difference in power is modest.

References

Benjamini, Y. and Hochberg, Y. (1995). Controlling the false discovery rate: a practical and powerful approach to multiple testing. *Journal of the Royal statistical society: series B (Methodological)*, 57(1):289–300.

Love, M. I., Huber, W., and Anders, S. (2014). Moderated estimation of fold change and dispersion for rna-seq data with deseq2. *Genome biology*, 15:1–21.

Mallick, H., Rahnavard, A., and McIver, L. (2020). Maaslin 2: Multivariable association in population-scale meta-omics studies. *R/Bioconductor Package*.

Mandal, S., Van Treuren, W., White, R. A., Eggesbø, M., Knight, R., and Peddada, S. D. (2015). Analysis of composition of microbiomes: a novel method for studying microbial composition. *Microbial ecology in health and disease*, 26(1):27663.

Ritchie, M. E., Phipson, B., Wu, D., Hu, Y., Law, C. W., Shi, W., and Smyth, G. K. (2015). limma powers differential expression analyses for rna-sequencing and microarray studies. *Nucleic acids research*, 43(7):e47–e47.

Robinson, M. D., McCarthy, D. J., and Smyth, G. K. (2010). edger: a bioconductor package for differential expression analysis of digital gene expression data. *bioinformatics*, 26(1):139–140.
